# Delayed gastric emptying after classical Whipple or pylorus-preserving pancreatoduodenectomy: a randomized clinical trial (QUANUPAD)

**DOI:** 10.1007/s00423-022-02583-9

**Published:** 2022-07-04

**Authors:** J. Busquets, S. Martín, Ll. Secanella, M. Sorribas, N. Cornellà, J. Altet, N. Peláez, M. Bajen, T. Carnaval, S. Videla, J. Fabregat

**Affiliations:** 1 Department of Hepatobiliary and Pancreatic Surgery, Bellvitge University Hospital, Research Group of Hepato-Biliary and Pancreatic Diseases, Institut d’Investigació Biomèdica de Bellvitge - IDIBELL, University of Barcelona, Carrer de la Feixa Llarga s/n, 08907 L´Hospitalet de Llobregat, Barcelona, Spain; 2 Departament de Ciències Clíniques, Facultat de Medicina i Ciències de la Salut, Universitat de Barcelona (UB), c. Casanova, 143, 08036 Barcelona, Spain; 3 General and Digestive Surgery Service, Viladecans Hospital, Viladecans, Spain; 4 General and Digestive Surgery Service, Mar Hospital, Barcelona, Spain; 5 Department of Nuclear Medicine, Bellvitge University Hospital, University of Barcelona, Carrer de la Feixa Llarga s/n, 08907 L´Hospitalet de Llobregat, Barcelona, Spain; 6 Clinical Research Support Unit (HUB·IDIBELL), Clinical Pharmacology Department, Bellvitge University Hospital, Carrer de la Feixa Llarga s/n, 08907 L´Hospitalet de Llobregat, Barcelona, Spain; 7 Pharmacology Unit, Department of Pathology and Experimental Therapeutics, School of Medicine and Health Sciences, IDIBELL, University of Barcelona, Carrer de la Feixa Llarga s/n, 08907 L´Hospitalet de Llobregat, Barcelona, Spain

**Keywords:** Delayed gastric emptying, Pyloric preservation, Classical Whipple, Pylorus-preserving pancreatoduodenectomy

## Abstract

**Purpose:**

Pylorus-preserving pancreatoduodenectomy (PPPD) has been the gold standard for pancreatic head lesion resection for several years. Some studies have noted that it involves more delayed gastric emptying (DGE) than classical Whipple (i.e., pancreatoduodenectomy with antrectomy). Our working hypothesis was that the classical Whipple has a lower incidence of DGE. We aimed to compare the incidence of DGE among pancreatoduodenectomy techniques.

**Methods:**

This pragmatic, randomized, open-label, single-center clinical trial involved patients who underwent classical Whipple (study group) or PPPD (control group). Gastric emptying was clinically evaluated using scintigraphy. DGE was defined according to the International Study Group of Pancreatic Surgery (ISGPS) criteria. The secondary endpoints were postoperative morbidity, length of hospital stay, anthropometric measurements, and nutritional status.

**Results:**

A total of 84 patients were randomized (42 per group). DGE incidence was 50% (20/40, 95% confidence interval (95% CI): 35–65%) in the study group and 62% (24/39, 95% CI: 46–75%) in the control group (*p* = 0.260). No differences were observed between both groups regarding postoperative morbidity or length of hospital stay. Anthropometric measurements at 6 months post-surgery: triceps fold measurements were 12 mm and 16 mm (*p* = 0.021). At 5 weeks post-surgery, triceps fold measurements were 13 mm and 16 mm (*p* = 0.020) and upper arm circumferences were 26 cm and 28 cm (*p* = 0.030). No significant differences were observed in nutritional status.

**Conclusion:**

DGE incidence and severity did not differ between classical Whipple and PPPD. Some anthropometric measurements may indicate a better recovery with PPPD.

**Trial registration:**

ClinicalTrials.gov Identifier: NCT03984734.

## Introduction

Pancreatoduodenectomy is the gold standard technique for treating the tumors of the periampullary area [1, 2]. Although the initial description (classical Whipple) included antrectomy, pyloric preservation has been successful by digestive surgeons in recent years. Advocates argue that it achieves lower blood loss and better quality of life (QoL) [3, 4]. However, subsequent studies showed that pyloric preservation might be associated with an increase in delayed gastric emptying (DGE) [5–9]. Conversely, meta-analyses [10–12] showed no differences between the incidence of DGE in pylorus-preserving pancreatoduodenectomy (PPPD) and classical Whipple. This issue is controversial and a matter of concern [13].

According to the International Study Group of Pancreatic Surgery (ISGPS) guidelines on nutritional support and therapy in pancreatic surgery, patients who undergo pancreatoduodenectomy should be carefully monitored to assess the presence of endocrine and/or exocrine pancreatic insufficiency and nutritional status [14]. However, to our knowledge, no data are available on anthropometric changes after pancreatoduodenectomy.

Our working hypothesis was that the classical Whipple has a lower DGE incidence. Therefore, we aimed to compare the incidence of DGE between pancreatoduodenectomies (classical Whipple or pylorus preservation) after surgery in this randomized clinical trial (RCT). Similarly, we aimed to provide comparative data on postoperative morbidity, nutritional status, and anthropometric measurements after pancreatoduodenectomy.

## Materials and methods

### Study design

This was a randomized (1:1), open-label, single-center, controlled, parallel-group, pragmatic clinical trial of patients who underwent pancreatoduodenectomy.

The study protocol was approved by the Ethics Committee (185/03) of the Bellvitge University Hospital, University of Barcelona, and it was registered at ClinicalTrials.gov (QUANUPAD Trial; NCT03984734). Written informed consent was obtained from all the patients. The study was conducted in accordance with the updated Declaration of Helsinki, guidelines for Good Clinical Practice, and applicable Spanish regulatory requirements. Confidentiality was guaranteed in accordance with current Spanish legislation (LOPD 15/1999—currently repealed, LOPD 3/2018). This manuscript was written in accordance with the CONSORT guidelines [15].

### Study population

This study included adult patients (≥ 18 years of age) of both sexes who underwent surgical partial pancreatoduodenectomy (pancreatic head resection) at the Bellvitge University Hospital and provided written informed consent. Exclusion criteria were as follows: (i) patients who underwent total pancreatectomy; (ii) patients who underwent incomplete pancreatoduodenectomy; (iii) patients who received associated resections of other organs, except for the portal or superior mesenteric veins; (iv) patients with previous gastrectomy or other gastric surgery; (v) patients receiving neoadjuvant treatment; (vi) patients with liver cirrhosis; and (vii) patients with duodenal ischemia or tumor infiltration that required an antrectomy.

The following anonymized data were entered into an ad hoc-created case report form (CRF): date of birth, date of diagnosis, date of surgery, anthropometric measurements (weight, upper arm circumference, tricipital skinfold), scintigraphy study of gastric emptying, nutritional status (analytical variables: liver enzymes, albumin, prealbumin, C-reactive protein (CRP)), readmissions, morbidity, and QoL.

### Randomization and masking

An external statistical consultant created a randomization list (1:1) before the start of the study. Patients were allocated to one of two study groups: (1) study group: patients who underwent classical Whipple, or (2) control group: patients who underwent PPPD.

The external statistician’s team, the only one with access to the randomization list, prepared envelopes (one per patient) containing the randomization code. They were opaque, sealed, and numbered sequentially. Randomization was performed by opening the envelope with a blinded assistant of the surgical team.

Randomization was performed before starting resection, as described in another study [16]. The randomization timing (before starting the resection) was selected to avoid including patients with exclusion criteria only detectable at the time of the surgery, such as (i) an incomplete pancreatoduodenectomy due to intraoperative findings (i.e., liver metastasis) and (ii) other intraoperative findings that require a change of course in the previously programmed surgical plan (e.g., total pancreatectomy due to an affected margin).

This was an open-label study in which both the patient and the surgeon knew which type of reconstruction was conducted. A blinded third-party evaluation of the primary endpoint was not performed.

### Study procedure

The study duration was from the surgery day (day 0, randomization) to 6 months after surgery. The patient was monitored daily from the surgery day until the day of hospital discharge. Outpatient control visits were scheduled for the first week after discharge and the 5^th^ week and 6^th^ month after surgery (day 0).

#### Surgical technique

All interventions were performed by a team of surgeons with experience in hepatobiliary and pancreatic surgeries. All surgeons specialized in hepatobiliary surgery and liver transplantation.

All patients underwent resection with curative intent, including partial pancreatoduodenectomy with standard lymphadenectomy [17, 18]. We started the surgery with exploratory laparoscopy to rule out previously undetected disease extensions. A right subcostal laparotomy was performed, followed by cholecystectomy with lymphadenectomy of the hepatic hilum. Duodenal or gastric transections were obtained using a stapler. Resection surgery was performed according to international cancer standards. In the study group, patients underwent classical Whipple, and distal gastrectomy varying from 20 to 40% was performed. In the control group, patients underwent PPPD; in these cases, the right gastric artery was preserved unless the artery restricted gastric mobility. The duodenum was dissected and divided at least 2 cm distally to the pylorus. Reconstruction was performed on a single loop, starting with pancreatic anastomosis. Pancreatic, biliary, and gastric anastomoses were performed in a retrocolic position. Duct-to-mucosa pancreatojejunostomy was the first choice for all patients. In the case of a narrow pancreatic duct, an internal transanastomotic pancreatic duct stent was introduced after pancreatojejunostomy at the surgeon’s discretion. Hepaticojejunostomy was performed approximately 15 cm from pancreatojejunostomy.

Gastric or duodenal anastomosis was performed depending on the randomization group. Retrocolic duodenoenteric or gastroenteric anastomoses were performed using silk sutures. In cases of duodenal ischemia at the time of suturing or massive periampullary tumors with possible duodenal affectation, the duodenum was divided, and a gastroenteric anastomosis was performed. These patients were excluded from the final data analysis. These anastomoses were performed approximately 60 cm from the biliary anastomosis site. Braun enteroenterostomies were not performed. In both groups, two drains were placed close to the pancreatojejunal anastomosis (*n* = 2) and one posteriorly to the hepaticojejunal anastomosis (Bellovac, Wellspect, HealthCare, Möndal, Sweden). After surgery, a nasogastric suction tube was placed in all patients.

#### Postoperative care

The analgesic treatment protocol and diet progression scheme during the postoperative period were identical between the two groups. During the first 24 h, the patients were monitored in an intensive care unit under the care of the anesthesia department. All patients were administered opioid- and non-steroidal anti-inflammatory drug-based analgesics, antiemetics every 8 h, and nasogastric aspiration. After 24 h, patients were transferred to the surgical ward, and analgesics were administered at the surgeon’s discretion. The nasogastric tube was maintained until the suction debit was less than 800 mL/day, and correct clamping tolerance was observed (i.e., with no clinical or radiological suspicion of gastric stasis). In cases of clinical doubt, a gastrointestinal transit study with oral contrast was performed, and the nasogastric tube was removed when there was evidence of contrast passage across the anastomosis. Patients were provided total parenteral nutrition if no oral tolerance was achieved by the 7^th^ day. Metoclopramide and ondansetron were administered every 6 h on an alternating regimen to all patients until the nasogastric tube was removed. In cases of fever or suspected sepsis, an abdominal CT scan was performed to rule out an intra-abdominal abscess. After removal of the nasogastric tube for at least 7 days, treatment with antiemetics was maintained until progressive withdrawal at the surgeon’s discretion. Amylase levels in the drainage fluid were measured on the first and third days after surgery. The drains were removed when amylase levels were less than threefold those in the blood. None of the patients received erythromycin or somatostatin [19, 20].

#### Follow-up

Before surgery (day 0), baseline characteristics, anthropometric measurements, nutritional status (laboratory variables—see the “Outcomes” section for more detail), scintigraphy study of gastric emptying, and QoL questionnaires were gathered. Study visits were scheduled during the perioperative period. Clinical follow-up of all patients was performed by the same team of surgeons. All patients visited the hospital twice during the first 90 days: the first week after discharge and the 5^th^ week after surgery. The follow-up was performed during outpatient visits by the surgeon in charge. In the 5^th^-week control visit after surgery, anthropometric measurements and nutritional status (laboratory variables) were recorded, and a scintigraphy gastric emptying study was performed. Data on readmission and morbidity at 90 days after surgery were obtained from the medical files. During the control visit at 6 months, nutritional status (laboratory variables) and anthropometric measurements were reobtained, and patients were required to answer a new QoL questionnaire.

### Outcomes

The primary efficacy endpoints were the incidence and severity of DGE. Gastric emptying was clinically evaluated using scintigraphy. DGE and its severity (DGE grade) were defined according to the ISGPS criteria [3, 21].

Scintigraphy of gastric emptying was performed according to the hospital’s protocol. In all patients, gastric emptying at orthostasis was measured before surgery and at 5 weeks post-surgery. The patients were radiolabeled with 1 mCi (37 Mbq) of ^99m^Tc-colloid mixed with eggs, two slices of bread, and 200 mL of water (ingestion time < 10 min). Following ingestion, gastric area images were obtained at 15-min intervals for 90 min using a single-head gamma camera equipped with a 140-keV high-resolution collimator. The gastric area and background regions of interest were outlined in the first image and projected on the following images, and the percentage of retained gastric activity (GR) at 90 min versus the baseline image was calculated [22]. Pathological gastric emptying (DGE) was defined as gastric retention according to scintigraphic criteria (isotope that remains in the stomach) in patients with a radiotracer percentage greater than 61% at 90 min [22].

The secondary endpoints were safety (postoperative morbidity), length of hospital stay, anthropometric measurements, nutritional status, and QoL.

Postoperative morbidity was defined according to the Clavien–Dindo classification [23]. Postoperative morbidity encompassed the appearance of any kind of morbidity during the hospital stay, and the final decision regarding its presence was made by consensus among the surgical team members after the daily visit. Perioperative mortality was defined as death during the same hospital admission and within 90 days after surgery if the patient was discharged early.

Information on whether the patient had a narrow pancreatic duct and/or a pancreatic fistula was also gathered. A narrow pancreatic duct was defined as a diameter ≤ 3 mm. A pancreatic fistula was defined as the presence of an outflow of amylase-rich drainage fluid after the 3^rd^ postoperative day and was classified according to the International Study Group for Pancreatic Fistula criteria [24].

Readmissions during the first 90 days after the surgery were recorded. Patients with postoperative death were excluded from the DGE analysis.

The anthropometric measurements used were weight, arm circumference, and triceps skinfolds. These were recorded in the preoperative period and at 5 and 6 months after surgery [25]. The upper arm circumference, assessed at the midpoint of the proximal arm (cm), was measured using a measuring tape. The tricipital skinfold (mm) was measured using a plicometer and a dermatograph pencil. In accordance with the Bistrian and Blackburn criteria [26], the average upper arm circumference values accepted were 24.3 cm in men and 17.7 cm in women. Regarding the tricipital skinfold, the values accepted were 12.5 mm in men and 22.3 mm in women.

Nutritional status was assessed using the following laboratory tests: liver enzymes, albumin, prealbumin, creatinine, urea, and CRP, at the preoperative visit and at the 5 weeks and 6 months post-surgery visits. All laboratory tests were performed according to the quality standards of the reference laboratory.

QoL was evaluated using the QLQ-PAN26 questionnaire [27], which explores different areas and assigns a value to each answer. A numerical result was obtained, and the higher the value, the worse the QoL. QoL was evaluated at the preoperative visit and at the 6-month postoperative visit.

### Statistical analysis

The sample size was calculated based on the incidence of DGE after pancreatoduodenectomy. The expected incidence of DGE after PPPD (control group) was 43% [5], and that after classical Whipple (study group) was 10% [5]. To detect differences in the contrast of the null hypothesis (H_0_: p1 = p2) using a two-sided *χ*^2^ test for two independent samples, with an *α* error of 0.05, a statistical power of 0.90, and a dropout rate of 10%, 40 patients were required in each group (1:1).

The data were encrypted and stored in a database created using Microsoft Access® (Microsoft, Redmond, WA, USA). The statistical analysis was only based on the “full analysis” set. Continuous variables are reported as mean (standard deviation (SD)). Variables that followed a normal distribution were analyzed using Student’s *t*-test for the comparison of means and the *χ*^2^ test (Fisher’s exact test for expected values < 3) for the comparison of proportions. The *χ*^2^ test or Fisher’s exact test was used to analyze categorical variables. The Mann–Whitney *U* and Wilcoxon *W* tests were used for variables that did not follow a normal distribution. *p*-value < 0.050 was considered statistically significant. Statistical analyses were performed using SPSS® software version 18 (IBM, Armonk, NY, USA).

## Results

### Baseline characteristics

**Fig. 1:**
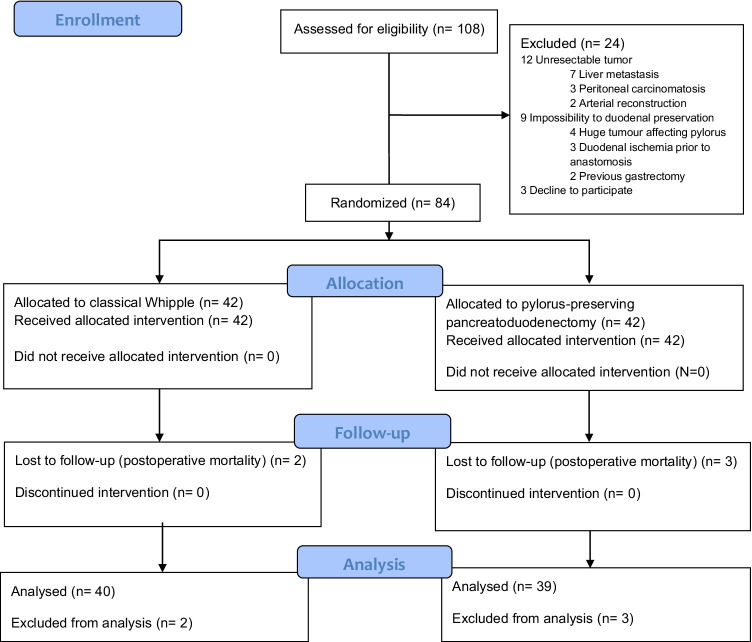
CONSORT 2010 flow diagram

A total of 108 patients were assessed for eligibility between August 2003 and August 2008. Of these, 24 were excluded for various reasons. Therefore, 84 patients were randomized (42 patients per group). Five patients died during the in-hospital postoperative period. These patients were not included in the analysis of DGE incidence; therefore, 79 patients were analyzed (40 in the study group and 39 in the control group). The flowchart of the study is shown in Fig. 1, and the baseline patient characteristics are shown in Table 1. The biliary tract was drained only in 15/84 patients (18%), with no differences between the two groups.

**Table 1:** Preoperative demographic and clinical characteristics and laboratory analyses of the patients included in the study

Variables	**Classical Whipple** **(study group)** (*N* = 42)	**PPPD** **(control group)** (*N* = 42)
Age (years), mean (SD)	63.9 (12.0)	66.3 (11.1)
Sex (male), *n* (percent)	24 (57.1)	25 (59.5)
Weight before the disease (kg), mean (SD)	73.8 (14.1)	79.9 (12.0)
Preoperative weight loss (kg), mean (SD)	7.9 (5.8)	7.4 (4.6)
Preoperative arm circumference (cm), mean (SD)	27.7 (3.6)	28.9 (3.6)
Tricipital skinfold (mm), mean (SD)	13.9 (7.3)	14.9 (6.5)
Diagnosis, *n* (%)		
Pancreatic ductal adenocarcinoma	24 (57.1%)	20 (47.6%)
Ampullary adenocarcinoma	6 (14.3%)	12 (28.6%)
Distal bile duct carcinoma	4 (9.5%)	4 (9.5%)
Neuroendocrine tumor	2 (4.8%)	2 (4.8%)
Chronic pancreatitis	3 (7.1%)	0
Intraductal papillary mucinous neoplasm	0	2 (4.8%)
Mucinous cyst neoplasm	1 (2.4%)	0
Stromal tumor	1 (2.4%)	0
Other	1 (2.4%)	2 (4.8%)
Preoperative bilirubin (µmol/L), mean (SD)	211.1 (185.9)	168.6 (176.0)
Preoperative glucose (mmol/L), mean (SD)	6.7 (2.9)	8.2 (10.9)
Preoperative triglyceride (mmol/L), mean (SD)	2.8 (1.2)	7.2 (31.7)
Preoperative hematocrit (%), mean (SD)	34.6 (4.7)	35.3 (7.1)
ASA physical status, *n* (%)		
II	21 (50.0%)	13 (31.0%)
III	21 (50.0%)	29 (69.0%)
APACHE II scoring system, mean (SD)	7.05 (3.4)	7.17 (2.8)

*PPPD*, pylorus-preserving pancreatoduodenectomy

### *Primary efficacy endpoint*

The incidence of DGE was 50% (20/40, 95% confidence interval (CI): 35–65%) in the study group and 62% (24/39, 95% CI: 46–75%) in the control group (*p* = 0.260).

DGE was associated with a longer hospital stay in both groups. The scintigraphy studies performed before surgery and at the 5^th^ week after the intervention showed a mean (SD) percentage of radiotracer retention at 90 min of 35% (2.7) and 41% (2.9), respectively.

No significant differences were observed between the groups regarding the percentage of radiotracer retention at the preoperative study (39.9% and 31.2%, respectively) or the 5^th^-week post-surgery study (42.6% vs. 39.6%). Finally, we did not observe significant differences when comparing pre- and postoperative scintigraphy studies according to different DGE degrees.

### Secondary efficacy endpoints

No differences were observed in the length of hospital stay between both groups (Table 2).

**Table 2:** Evolution and postoperative morbidity

Variable	Classical Whipple (study group) (*n* = 40)	Pylorus-preserving partial pancreaticoduodenectomy (control group) (*n* = 39)	*p* value
Delayed gastric emptying, ISGPS criteria; *n* (%)	20/40 (50%)	24/39 (61.5%)	0.302
Grade of delayed gastric emptying; ISGPS criteria, *n* (%)			
Grade A	9 (22%)	9 (23.1%)	
Grade B	4 (10%)	10 (25.6%)	
Grade C	7 (17.5%)	5 (12.8%)	0.307
Nasogastric tube required (days; mean (range))	11.56 (1–22)	10.86 (4–15)	0.811
Unable to tolerate liquid oral intake by postoperative (day; mean (range))	13.32 (2–24)	12.64 (6–19)	0.815
Percentage of radiotracers at 90 min (mean ± sd); 5^th^-week isotopic test	42.6 ± 4.6	39.6 ± 4.1	0.684
Radiotracer percentage greater than 60% in the study at 90 min; 5th-week isotopic test	8/38 (21.1%)	4/36 (11.1%)	0.246
Duct-to-mucosa pancreaticojejunostomy, *n* (%)	32 (76%)	28 (66.7%)	0.370
Perioperative transfusion (< 48 h) (yes), *n* (%)	20 (47.6%)	13 (31%)	0.282
Duration of operation (min), mean (range)	378.81 (311.5–446.12)	380.48 (298.96–462)	0.919
Morbidity, *n* (%)	25/42 (59.5%)	27/42 (64.3%)	0.653
Medical complications	10 (24%)	12 (26.6%)	0.860
Pancreatic fistula, *n* (%)	3 (7.1%)	8 (19%)	0.109
Postoperative hemorrhage, *n* (%)	6 (14.3%)	2 (4.8%)	0.137
Intraabdominal abscess, *n* (%)	7 (16.7%)	8 (19%)	0.776
Biliary fistula, *n* (%)	2 (4.8%)	0	0.152
Gastrointestinal fistula, *n* (%)	2 (4.8%)	2 (4.8%)	1
Reoperation, *n* (%)	3/42 (7.1%)	5/42 (11.9%)	0.457
Hospital stay (days; mean ± sd)	21.98 ± 17.78	22.36 ± 12.98	0.514
Mortality, *n* (%)	2/42 (4.8%)	3/42 (7.1%)	0.645
Quality-of-life assessment (QLQ-PAN26)	59.67 (2.0)	58.45 (2.2)	0.379

Regarding the anthropometric measurements, the weight before the disease was lower in the study group. Progressive weight loss was observed in both groups from the beginning of the disease until the 6^th^ postoperative month. The upper arm circumference at 5 weeks post-surgery was greater in the control group than in the study group (26 cm vs. 28 cm, *p* = 0.030). The triceps fold measurement was greater in the control group at the 5^th^ week (13 mm vs. 16 mm, *p* = 0.020) and 6^th^ month (12 mm vs. 16 mm, *p* = 0.021) post-surgery than in the study group.

No significant differences were observed in the analytical values related to nutritional status.

The QoL questionnaire QLQ-PAN26 did not show differences between both groups either preoperatively or 6 months after surgery. During the 6-month follow-up period, 41 patients received chemotherapy, with no differences between both groups (*p* = 0.162). Additionally, 38 patients received radiotherapy, with no significant differences between both groups (*p* = 0.251) (Table 2).

### Safety endpoint

The postoperative mortality rates were 4.8% (2/42) and 7.1% (3/42) in the study and control groups. Two patients died due to hemoperitoneum, two due to intra-abdominal sepsis and residual pancreatitis, and one due to acute myocardial infarction. No significant difference in mortality was observed between both groups.

Of 84 patients, 45 (53.6%) developed morbidity. However, no difference in overall morbidity was observed between both groups.

A greater incidence of pancreatic fistula was recorded in patients with invaginated anastomosis than in those with duct-to-mucosa anastomosis (17% vs. 12%). In addition, postoperative bleeding (percentage) was higher in the study group than in the control group; however, the difference was insignificant.

The percentage of reoperations was higher in the control group than in the study group; however, the difference was insignificant. Eight (10.1%) of 79 patients were reoperated on: three in the study group and five in the control group.

## Discussion

Although the incidence of DGE was lower in patients who underwent classical Whipple (50%) than in those who underwent PPPD (62%), this difference was not significant.

Nevertheless, the incidence observed is remarkable, which we could justify for several reasons. First, during the study period, we adopted a more conservative approach to postoperative care. Thus, the nasogastric tube was maintained until 24–48 h after surgery and was removed only if the gastric aspirate was low. Second, since 2010, we have been performing antecolic gastrojejunal anastomoses [28], which has led to an improvement in DGE in these patients. Therefore, we are currently more active in initiating oral feeding, and we have also incorporated the Enhanced Recovery After Surgery (ERAS) program as part of our usual clinical practice to shorten postoperative hospital stay [29].

As mentioned above, no differences in DGE were observed when the two techniques were compared. However, several reflections must be made. In our study, we were unable to safely complete pancreatoduodenectomy with pylorus preservation in nine patients, and a classical Whipple had to be performed. Some patients had massive tumors, jeopardizing the oncological acceptability of resection. In other cases, the risk of duodenal ischemia forced us to perform gastrectomy. Similarly, Lin et al. [5] performed gastrectomy on five patients, and Tran et al. [30] on two. Tran et al. also found a higher percentage of affected tumor margins in the PPPD group than in the classical Whipple group (19 (26%) vs. 12 (17%), *p* = 0.023). Regarding these differences, there was a higher percentage of peripancreatic circumferential margins, defined as the dorsal resection margin (peripancreatic fat and fascia of Treitz) or beyond the anterior pancreatic parenchyma anteriorly (peripancreatic fat, mesenteric base of the transverse colon, or posterior peritoneum of the lesser sac) in the PPPD group when compared to the classical Whipple group, and one patient presented an affected duodenal margin. In addition, the PPPD group had a higher number of resections with affected margins [30]. Thus, in our opinion, the classical Whipple technique should be adopted as the first choice since it has the same morbidity rate and can be performed in all patients without the fear of duodenal ischemia. In addition, the risk of a positive margin in pylorus preservation should be considered.

RCTs published to date do not help decide which of these two techniques is better at reducing DGE incidence. In the late 1990s, Paquet et al. [7] and Wenger et al. [31] showed that PPPD was superior to the classical Whipple procedure in terms of better nutritional and endocrine recovery, and postoperative QoL. However, in 1999, Lin et al. [5] reported a higher incidence of DGE in patients who underwent PPPD. Subsequently, in the 2000s, Tran et al. [30] and Seiler et al. [32] published similar results between the two techniques in terms of postoperative morbidity and DGE. In 2008, Srinarmwong et al. [8] reported that PPPD was associated with a higher incidence of DGE. Finally, in 2015, Taher et al. [33] reported similar morbidity rates between the two techniques. Meta-analyses comparing the classical Whipple procedure and PPPD [34–38] also failed to demonstrate a clear difference in DGE incidence between the two techniques.

In recent years, several groups have advocated pyloric ring resection as a measure to improve DGE. Several RCTs [39–42] and meta-analyses [36–38, 43, 44] that compared pyloric ring preservation with resection have been published. The last two meta-analyses comparing pyloric ring resection and preservation in pancreatoduodenectomy indicated that pyloric resection is superior to pyloric preservation in terms of DGE [37, 38]. However, evidence in this regard is unclear.

The debate over a technique that provides the least DGE is still ongoing; the proof of this is the publication of two recent articles based on German and American data. In this sense, a German study on DGE risk factors was based on a record of more than 5000 patients [45] without being able to demonstrate differences between the two techniques. Notably, PPPD was the method of choice in most patients (70.4%). A recent publication of a large American study of more than 15,000 patients [46] revealed the need for continued investigation of the factors responsible for greater DGE. The authors created the PrEDICT-DGE score to identify patients at high risk for DGE and help guide perioperative management. Some procedures, such as concurrent adhesion, feeding jejunostomy, vein graft vascular reconstruction, or pancreatic invagination anastomosis, were identified as independent factors associated with DGE. Based on their findings, the author suggested that classical Whipple with duct-to-mucosa pancreatojejunostomy technique should be considered the primary surgical approach in high-risk DGE patients.

### Nutritional status

The patient’s nutritional status and risk assessment of postoperative malnutrition should be part of the usual clinical practice before any pancreatic surgery, as recommended by the ISGPS [14]. The available data do not show any definitive nutritional advantages for a specific type of gastrointestinal reconstruction technique after pancreatoduodenectomy [42, 47, 48]. Therefore, a preoperative evaluation of patients undergoing pancreatic surgery should be performed, incorporating, among others, the percentage of body weight loss over time and body mass index (BMI) [40]. However, to date, no other group has investigated anthropometric changes after pancreatic surgery in relation to the type of gastroenteric reconstruction. We designed an RCT to evaluate postoperative DGE and analyze the nutritional status of patients after pancreatic surgery, with reference to pyloric preservation.

Maintaining appropriate nutritional support in patients with acute and chronic illnesses is a fundamental part of standard medical and surgical care. Malnourished patients have poorer clinical outcomes and higher morbidity and infection rates, and demand more healthcare resources than well-nourished patients [49]. Gastroparesis is one of the most under-diagnosed problems in patients with cancer and is often overlooked as a potential etiology of chronic nausea and vomiting. The exact prevalence of DGE is unknown; however, it is generally recognized that gastroparesis is common among patients with upper gastrointestinal tract tumors [50, 51] and after surgical treatment. A diagnosis of DGE is important in cancer and postoperative patients because the consequences of malignancy-associated gastroparesis can be serious, particularly in the context of other common problems that affect nutrition and fluid-electrolyte balance. Weight loss, anthropometric measurements, and various analytical variables have been associated with postoperative morbidity [14, 52–55]. The 5^th^-week postoperative evaluation showed superior anthropometric measurements in the PPPD group than in the study group in both the upper arm circumference and triceps fold (Fig. 2). However, at 6 months, these differences persisted only for the triceps fold measurement. We did not find a valid explanation for these differences; anthropometric changes likely respond to small modifications in the body constitution, which could result from the surgical technique. However, future studies should assess the anthropometric changes during the postoperative period of pancreatoduodenectomies. There were no differences in weight loss, BMI, or any of the laboratory variables analyzed between the study groups.

### Isotope study of gastric emptying

Currently, gastric emptying scintigraphy following a standardized solid meal or liquid-phase gastric emptying (with ^99m^Tc-radiolabeled pertechnetate mixed in orange juice) is the gold standard for diagnosing gastroparesis [25]. It can also be used to monitor the effectiveness of prokinetic therapy, although repeated exposure to radiation may be a limitation [56, 57]. Isotope studies after pancreatoduodenectomy have been used for years to assess DGE after pyloric preservation [57–61]; however, they have never been used in an RCT comparing both pancreatoduodenectomy techniques.

Although some authors [58] observed no differences in scintigraphy between both types of pancreatoduodenectomies, others [59] observed that more severe cases of DGE were associated with a higher percentage of residual radioactivity in the stomach after 120 min. Additionally, a more recent study [60] showed that scintigraphy performed on the 10^th^ day after pancreatoduodenectomy had a better correlation with clinically relevant DGE than scintigraphy performed on the 21^st^ day. In this study, we performed scintigraphy preoperatively and at 5 weeks postoperatively to observe long-term changes in gastric emptying. We did not observe differences in the retention of radiotracers between the study groups, nor did we find greater retention of radiotracers in patients with DGE. However, we observed a higher percentage of radiotracers in patients with severe DGE, although it was not significant.

### Limitations

The first limitation of this study is the amount of time elapsed since we obtained our data, given that the analysis and presentation of the results were completed in 2010. Surgical practice and postoperative care have changed over the past 10 years. However, the surgical details of resection and reconstruction are essentially the same, and the postoperative morbidity remains similar. Furthermore, the study design followed the scientific method transparently and rigorously. Since new DGE concepts have been defined, and in light of recent publications that presented this problem regarding the magnitude of gastric resection, we thought it was necessary to report our results, which, despite being old, are reliable. As in other relevant studies, the timelessness of the problem is obvious, and, as previous meta-analyses advise, more RCTs should be performed to elucidate this. Second, the surgeons were responsible for the patients’ postoperative care. Masking was not possible because the investigators knew each patient’s randomization group. Given the results of this study, it does not seem necessary to preserve the pylorus during pancreatoduodenectomy since it did not provide any additional benefit. A long-term evaluation of the effect of PPPD was not performed and may be an endpoint to be pursued in future studies. Furthermore, a higher percentage of these patients died during the follow-up, making the long-term effect difficult to analyze. Another drawback is that the QoL PAN-26 results cannot be presented in detail because only the total score was collected in the database.

## Conclusion

The incidence and severity of DGE did not differ between patients who underwent classical Whipple and those who underwent PPPD. Some anthropometric measurements may indicate better recovery with PPPD; however, both surgical pancreatoduodenectomy techniques were similar in terms of morbidity and mortality, nutritional status, and QoL. Therefore, in our opinion, the classical Whipple procedure should be the technique of choice because it can be performed in all patients without the fear of duodenal ischemia.

## Data and/or code availability

The data sets used and/or analyzed during the study will be available from the corresponding author upon reasonable request.
